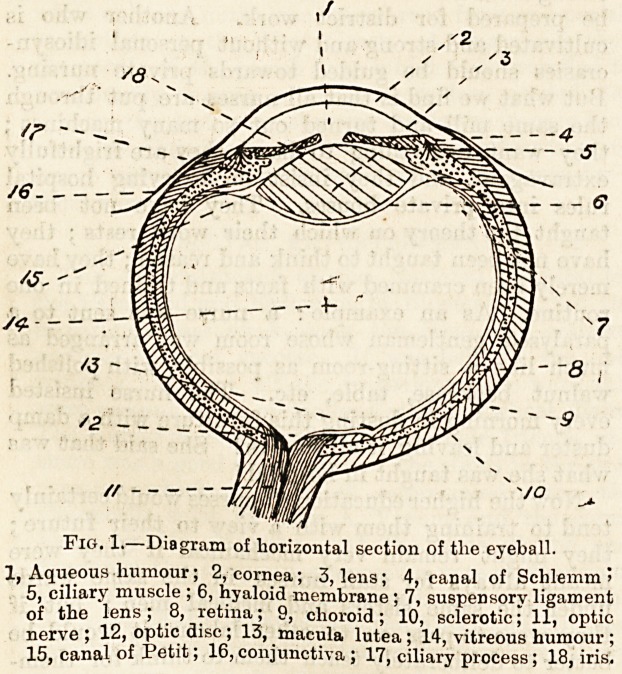# The Hospital. Nursing Section

**Published:** 1903-01-24

**Authors:** 


### The Hospital.

### Nursing Section.

Contributions for this Section of "The Hospital" should be addressed to the Editor, "The Hospital" Nursing Section, 28 & 29 Southampton Street, Strand, London, W.C.

No. 852.—Vol. XXXIII. *SATURDAY, JANUARY 24, 1903.*

### Notes on News from the Nursing World.

### THE QUEEN'S NURSES.

It will be seen from the official statement in another column that of the new Queen's nurses whose appointment her Majesty Queen Alexandra has been graciously pleased to approve, 64 are serving in England, 5 in Wales, 16 in Scotland, and 11 in Ireland. This is only the usual accession to the ranks at the beginning of the year, but there will doubtless be a large augmentation as soon as the Council of the Jubilee Institute are in a position to make use of the handsome sum subscribed to the fund for the Women's Memorial to Queen Victoria.

### THE MEMORIAL AGAINST THE CREATION OF THE "QUALIFIED NURSE."

We print to-day the copy of a memorial presented under the auspices of the Workhouse Infirmary Nursing Association to the President of the Local Government Board, which demands, and we are sure will receive, his most careful attention. It puts the case against the creation of a new grade of nurse under the title of "Qualified Nurse" in the clearest possible light and in the most conclusive manner. As it follows on the lines taken up on the subject in The Hospital we need not say more on this point. The memorial is signed by the Princess Christian, by the flower of the nursing profession, and by many eminent doctors, and hospital and other experts. The number of signatures of matrons of leading training schools only is nearly 60, of whom 33 are matrons of union infirmaries. In fact, the document may be shortly described as a manifesto from the nursing world against the recommendation of the Departmental Committee, which, as we said on the morrow of its publication, would, if carried into effect, degrade the standard of nursing. A supplementary memorial on the part of the Executive Committee of the Workhouse Infirmary Nursing Association also merits the favourable consideration of Mr. Long; but for the moment the interest of nurses will centre in the reception of the other document the significance and importance of which everyone will understand.

### THE PROBLEM SOLVED IN LANCASHIRE.

It appears from the report of Mr. Jenner-Fust on Poor Law administration in 1901 and 1902 that the difficulty of obtaining nurses has not been much felt in Lancashire. At about half the workhouse infirmaries probationers are in training, and a supply of charge nurses is thus forthcoming. Mr. Jenner-Fust says that where the selection of probationers is left in great measure to the superintendent nurse and the medical officer, as is the case in some of the largest and best administered infirmaries, the superintendent has generally a numerous list of applicants from which to select. The comfort of nurses, he declares, is "much more considered than formerly, and separate nurses' homes, or good accommodation in one administrative block, are becoming general. Of separate nurses' homes there are already ten in Lancashire, and several more are in prospect." He also points out, and enforces his contention by means of a tabulated statement, that the number of patients a nurse is expected to attend upon has greatly diminished. He affirms that this, "together with the more frequent provision of an adequate staff of night nurses, and of stated hours of recreation, has rendered the life of a nurse less irksome and more popular." We are not surprised that Mr. Jenner-Fust, speaking in general terms, maintains that the larger the infirmary the better the accommodation, and the less monotonous and more interesting the employment. He adds: "It is not to be wondered at that at a workhouse where one person is expected to attend by day and by night upon 50 patients, or even more, the Guardians should be unable to retain the services of a nurse, even though a liberal salary be offered; but usually, if Guardians will provide proper accommodation and an adequate staff, and will offer reasonable salaries, they will not have much difficulty in securing nurses."

### THE VACANCY AT JOHANNESBURG HOSPITAL.

The post of nursing superintendent at Johannesburg hospital is vacant, and applications from candidates should be sent at once to the Chairman of the Hospital Board, Box 1,050, Johannesburg. The salary offered is £300 a year, with board, lodging, and washing. First-class passage will be paid, and the successful applicant will be required to enter into an engagement for three years. In the light of Mr. Chamberlain's statement on Saturday, that the excessive cost of living in Johannesburg will shortly cease to be a factor in the situation, the terms offered by the Board are sufficiently attractive to ensure a number of applications. The Johannesburg Hospital was founded in 1888. There is a sister-matron, a staff of 27 nursing sisters, 14 lay nurses, and 18 probationers. The mixture of the religious and lay elements has not prevented the adoption of the system of three years' training, and the nursing superintendent has, of course, the control of the school.

### SPANISH-AMERICAN WAR NURSES.

The third annual meeting of the Spanish-American War nurses was held at Washington last month. A reference by Inspector Boyd, of the United States Navy, to the proposed maximum age for admission of nurses to the navy as 30 years, provoked many remonstrances on the ground that the limit was too low. One nurse, however, declared that it was a matter of no consequence, "since no nurse was ever over thirty." The proceedings generally on the occasion may be described as a combination of the practical and the recreative, one of the most interesting features at the meetings being a speech in which Mrs. Brinton, president of the Civil War Nurses' Association, drew a comparison between the work rendered so long ago and that of the younger nurses of the Spanish-American War. Subsequently, Mrs. Foster, an officer of the Spanish War Veterans' Auxiliary, described her visit as a delegate to the Red Cross Conference at Russia, and said that training in that country is practically unknown.

### BRAVE NURSES AT TYRONE COUNTY HOSPITAL.

Two members of the nursing staff at Tyrone County Hospital, Armagh, have been presented with gold medals suitably inscribed for having, at great personal risk, saved the life of a male patient who attempted to throw himself from the roof of the hospital. Dr. E. C. Thompson, M.P., surgeon of the institution, in asking the chairman, Canon Scott, to make the presentation, said he had not heard of a braver action in recent years; and the chairman declared that he did not believe that anyone who had earned the Victoria Cross had done greater service to their cause and country than these two brave nurses. "By their act," he added, "they have brought honour on the institution." We entirely agree with this opinion and also with the chairman's remark that the act shows the high standard of duty which obtains in the hospital, and reflects credit on the matron.

### GLASGOW NURSES' GUILD AND CLUB.

On Friday last the annual meeting of the Glasgow Nurses' Guild and Club was held in Park Church Hall. There was a good attendance of clergymen and others interested in the Guild, but the small turn out of nurses was disappointing. The Rev. Dr. Donald Macleod presided, and in a résumé of the year's work said it was gratifying to see how successful the Guild had been, the bedrooms had been fully occupied, two At Homes had been held, and meetings had taken place in the Western and Victoria infirmaries. The aim of the Guild is to provide opportunities for religious and social intercourse amongst nurses. The club is well supplied with papers, books, and magazines, and many letters have been received from nurses who had stayed there saying how comfortable they had been. He congratulated the ladies' committee on the balance over of £12 8s. Dr. Eliz. Pees moved the reappointment of the committee, and thought that they deserved great praise for the trouble they had taken in collecting so many subscriptions. After such a good start has been made, it is hoped that nurses will show their appreciation of the work and kindness of the committee, and other friends by joining the Guild. At present there are 200 members.

### NURSES AND GOSSIPING.

In no profession is "a still tongue" of more value than that of a nurse. But it is only fair to recognise that, while it is a clear duty to refrain entirely from gossip, there are frequently temptations to indulge in it. At the annual meeting of the Coventry Nursing Institution Dr. Milner More said it was stated that people often hesitate to send for private nurses, and one reason given was that they gossiped. His rejoinder was that through the whole course of their training they were taught not to talk, but they were sometimes "encouraged" to gossip by ladies whom they attended. He might almost have said "invited." Dr. More confessed that it was often difficult for medical men to get out of speaking of cases. Of course, neither encouragement nor incitement to gossip is any justification for it on the part of nurses, but patients and their friends who do not check any tendency in that direction contribute to the offence.

### AMELIA SACH NOT A CERTIFICATED NURSE.

The woman Amelia Sach, who in conjunction with Annie Walters was last week convicted at the Old Bailey of wilful murder of an infant child, was described in most of the papers as "a certificated nurse." The last so-called "nurse" who appeared in a criminal court was the charwoman in the Chapman case who came forward as a witness. On this occasion although the use of the word "certificated" happened to be correct, the word "nurse" was quite inapplicable. Amelia Sach is a certificated midwife only, and there was the less excuse for any mistake in the description, as Mr. W. H. Leycester, by whom she was defended, quite correctly referred to her as such at the trial.

### CHELSEA INFIRMARY NURSES' LEAGUE.

The nurses of Chelsea Infirmary discussed on Friday evening in the recreation room the question of "State registration." The matron, Miss E. Barton, was in the chair, and several friends accompanied the nurses, of whom about forty were present. Mrs. Bedford Fenwick introduced the subject, and it was asked by the matron whether the proposals to grant certificates to infirmary nurses of one year's training was not an argument in favour of registration and the appointment of an independent advisory board. The reply was that if such a board had existed, no such reactionary measure would have been proposed, since public bodies dealing with nursing matters would naturally refer it to the board of experts, and act on the advice given. One of the nurses said she was certainly in favour of State registration, but she wanted to know whether if a register were made nurses who had only received a short training, but who had been a long time recognised as nurses, would be excluded. To this it was answered that no Parliamentary measure would be likely to be retrospective, and that the fairest mode of dealing with the question would be the allowance of years of grace as in the case of the Midwives Act, and the exclusion of such nurses would be highly improbable. Miss Bray said the only way to obtain State registration was to get a Parliamentary inquiry into the whole nursing question; tinkering was no good. A blue book on the nursing question would be a tremendous affair, but she did not think that much progress would be made without it. A Royal Commission would naturally be followed by a Bill.

### THE IMPORTANCE OF SLEEP.

The Guardians of the Newport Union, in Shropshire, do not appear to thoroughly realise the importance of sleep to the nurses in their employ. The bedroom of the nurses is over the room of an imbecile patient, and they complain that she prevents them from obtaining sleep. The Guardians talk about getting the doctor to certify for the removal of the patient to the asylum, but if it should be found that the case is not suitable for the asylum no action seems to be contemplated. The obvious duty of the board, however, is to make such arrangements that neither imbeciles, nor any other persons, can hinder the nurses from enjoying their rest undisturbed. Unless this is done it cannot be expected that they will maintain their health.

### EXCHANGE NO ROBBERY.

A statement that the managers of the Sutherland Benefit Nursing Association are removing nurses from thinly-populated and poorly-paying parishes in order to make use of their services in the flourishing parishes of the East Coast of Scotland is not, we are glad to learn, correct. The rumour may have had its origin in the fact that a nurse of a certain parish, where she is little employed, is to be transferred for a short time elsewhere in order to give several nurses holidays, or rests, on account of their being somewhat out of health. The transference of a nurse from one parish to another as occasion may require is also, we understand, in accordance with the constitution of the Association and is often done. From the beginning of November to January 10th there have been five instances of the kind, and of these four have been in the parishes of the East Coast, while only one nurse was transferred from one parish to another on the West Coast. As it appears that the people of the parish from which it is intended to temporarily remove a nurse so seldom needed her that in the last year she was employed in only four cases, we imagine that it will be an advantage to her to have rather more work and experience for a short time.

### HOSPITAL ENTERTAINMENTS.

The entertainment at the National Hospital for the Paralysed and Epileptic, in Bloomsbury, for the in-patients last week, took the form of a concert, and was a great success. It was held in the outpatients' hall, and all the in-patients who were able were present, some on trolleys, others in wheel chairs. The nurses were allowed to invite their friends, and there was a good attendance. The items in the programme which were most appreciated were the violoncello solos by Miss A. Vernet and the comic songs by Mr. Murphy. At the close of the performance the chaplain proposed a vote of thanks to the matron. From four to seven o'clock on Thursday last week the little patients in the Alexandra Hospital for Hip Disease were entertained by Punch and Judy, marionettes, and a conjurer. A large number of ladies and gentlemen visited the hospital and helped to amuse the children in the intervals. The wards were bright with flowers, and chains of fairy lamps suspended across the ceiling gave a very festive appearance to the scene.

### MEATH WORKHOUSE NURSING ASSOCIATION.

The Countess of Meath, presided at the last meeting of the committee of the Meath Workhouse Nursing Association, which was held at 83 Lancaster Gate. It was then decided to give certificates to those nurses who have remained in Poor Law appointments for at least one year. These certificates have since been presented and have given great satisfaction to the recipients. Some of the nurses have held the same Poor Law appointments for five years and many of them for three years. Lady Meath has spent much on premiums, uniforms, and pocket money, and the result must be gratifying to her. We understand that she is willing to accept more probationers for training. All applications should be made to the Hon. Secretary, Miss Lee, 51 Upper Baker Street, N.W., at the office of the Association, which gives a training of either one, two, or three years in a home, or a hospital, to candidates who undertake to become assistant-nurses in a workhouse after the training is completed.

### THE SCHOOL NURSE IN NEW YORK.

The school nurse system was adopted in New York last month, and seven nurses were at first appointed by the Commissioner of Health. He has since increased the number to twelve. The official definition of the duties of the school nurses is that they are to supplement the work of the medical inspectors by giving attention to such children as the inspector shall designate, by looking up the excluded children in their homes, and instructing the mothers. New York is the first city in the United States to introduce this useful and excellent system, but it may safely be concluded that other cities in America will speedily follow suit.

### A FOOTBALL MATCH FOR A NURSING INSTITUTE.

At Welshpool there has been a football match in aid of the funds of the Nursing Institute. The teams represented the football club committee and the licensed victuallers of the town respectively. To increase the interest of the public there was a procession to the field, and the services of the yeomanry band were requisitioned to lead it. The procession included the rival teams, and also upon a lorry was a tableau representing the work of the institute. A patient was seen in bed with a nurse sitting by his side, and at the front end of the lorry a banner was erected, upon which was inscribed the timely admonition, "Support your Nursing Institute." During the progress of the match a number of collectors went round with boxes, and a sum exceeding £10 was realised.

### DEARTH OF PRIVATE NURSES AT HEREFORD.

The governors of the Herefordshire General Hospital have been occupied with the question of the increase of the private nursing staff attached to the institution. Dr. Chapman cited as an illustration of the dearth of private nurses in Hereford that he had several times been called upon to wire to Gloucester, Cheltenham, Shrewsbury, and even to London for them, and he strongly urged that additional accommodation should be provided at the Herefordshire Hospital in order to enable the governors to augment the private nursing staff. Ultimately, a resolution in favour of the increase, and of the provision of further accommodation, was adopted, and a committee appointed to draw up a sheme to be considered at the next annual meeting. At present the number of private nurses varies from eight to 10, and it is contended that there is ample work in the city for 16 always, and sometimes for 20.

### The Nursing Outlook.

"From magnanimity, all fear above;
From nobler recompense, above applause;
Which owes to man's short outlook all its charm."

### HIGHER EDUCATION.

THOUGH the training of nurses in many of the best hospitals is very good, and though it is improving in many of the smaller schools, it is certain that America has outstripped us in this respect, and that whereas in the early days they had to send their nurses over here to be taught, we could now with advantage send a nurse to study American methods. The preliminary training, first started by the London hospital here, is now common in America, and New York is thinking of starting a "central school" where all probationers can spend their first three months. We shall not do that here until we also do away with our exclusive railway carriages, and have only corridor trains. There are probably no two matrons in London who would agree as to exactly what should be included in the first three months' instruction of the would-be nurse. But a little more pressure, a little more thought, might make several hospitals adopt the system of preliminary training, and not permit such woefully ignorant young women to commence their ministrations on the patients. This good commencement is the necessary basis for the good conclusion. Those who believe in the higher education of nurses, believe that many useless women get weeded out in these trial months, and that the useful women get started on the right lines from the very beginning. During the period of work in the wards theory should go hand in hand with practice. In a "course of study" drawn up by Miss Fisher of New Haven and Miss Glenn of Illinois, the "junior period" includes eight surgical lectures, five gynæcological, three contagious diseases, seven medical, six obstetrical, five children, two nervous system, and one on eye and ear; besides this there is demonstration or laboratory work in materia medica and so on. The examinations are to be as follows:—

"At the end of the freshman period, three: dietetics, bacteriology, practical work.

"At the end of the sophomore period, one: physiology and anatomy.

"At the end of the junior period, six: medical nursing, including care of contagious and nervous diseases; surgical and gynæcological nursing; obstetrical nursing, to include care of infant; nursing in diseases of the eye, ear, nose, and throat; materia medica; urinary analysis.

"At the end of the senior period, none.

"Markings will indicate standing in theoretical and practical work as well as in general conduct: A, excellent; B, good; C, fair; D. poor; F, failure.

"A public practice demonstration to be given annually by six members of the graduating class having the highest standing.

"In outlining this work, it has not seemed wise to arrange time for gymnasium exercises, but as it could be brought about this work would be introduced."

At Waltham Training School (U.S.A.) there are eight paid instructors of nursing. Now the value of examinations is slight; they are merely a useful adjunct to hospital teaching, and no amount of parchment can make a successful nurse. It is indeed doubtful in England whether we are not already getting rather tired of the highly-trained nurse for private work, and the frequent request to a doctor now is "May we not have in some useful woman? We have suffered so much from the lady nurse."

This is the result, not of too much training, but of want of elasticity and specialisation in the training, and the study of character. Far and away beyond all certificates is this formation of character by means of work in the wards; training in neatness and method, training in unselfishness and sympathy, training in patience and happiness. How many matrons really study and try to form the characters of their nurses? And how many who know and help their nurses have the common sense to vary the training according to the nurses' gifts? One woman may have special gifts in organising and should be taught to aim at a headship. Another with tact and strong faith in the brotherhood of mankind should be prepared for district work. Another who is cultivated and strong and without personal idiosyncrasies should be guided towards private nursing. But what we find is that all nurses are put through the same mill and turned out so many machines; they want everything to hand, they are frightfully extravagant, and they insist on carrying hospital rules into private houses. They have not been taught the theory on which their work rests; they have not been taught to think and reason; they have merely been crammed with facts and trained in one routine. As an example: a nurse was sent to a paralysed gentleman whose room was arranged as much like a sitting-room as possible, with polished walnut bookcase, table, etc. The nurse insisted every morning in dusting this furniture with a damp duster and leaving it all smears! She said that was what she was taught in hospital!

Now the higher education of nurses would certainly tend to training them with a view to their future; they might remain very mechanical if they were meant always for staff nurses in the same wards under the same sisters and medical men; but if they were to pass on to other labours it would be better to deliberately teach them to think for themselves, and tell them what to expect. The course at Columbia College (U.S.A.) described a fortnight ago, includes visits to different institutions. The course of hygiene at Bedford College, and the course for sanitary inspectors at the Sanitary Institute, include such visits also; but does any hospital at present send its nurses out to see what is being done elsewhere? Surely, if it did, there would not be such complaints of the absence of the most elementary knowledge of hygiene shown by district nurses. Let it be understood that if we wish to keep the place made for us by Florence Nightingale as head of the nursing world we must ever go on altering and improving, and must not think that "whatever is, is right."

### Lectures on Ophthalmic Nursing.

By A. S. COBBLEDICK, M.D., B.S.Lond., Senior Clinical Assistant Royal Eye Hospital, late House-Surgeon and Registrar, Royal Eye Hospital.

### LECTURE II.—THE CONTENTS OF THE ORBIT—ANATOMY OF THE EYEBALL.

*The Eyeball.*—Its shape is spherical, but it is not a true sphere on account of the fact that the anterior corneal portion —forming about one-sixth of the eyeball—is a segment of a smallar circle than is the posterior five-sixths. It is surrounded by a thin membrane—the capsule of Tenon—which isolates the ball, and allows of free movement with a minimum of friction, as a small amount of lubricating secretion is formed between the two. The capsule is attached to the optic sheath behind and to the sub-conjunctival tissue near the margin of the cornea.

*Structure.*—It is composed of three layers which enclose the refracting media. The layers are :—

1. *The sclerotic coat* which is pearly white in colour and commonly spoken of as the white of the eye. Anteriorly, this is continuous with the cornea, which is a clear transparent window. The point where the sclerotic passes into the cornea is well defined and is known as the corneoscleral junction.

2. *The choroid coat* which is separated from the sclerotic by a large lymphatic space, the perichoroidal space. Anteriorly this layer passes into the ciliary body and iris, to be hereafter described.

FIG. 1.—Diagram of horizontal section of the eyeball.

1, Aqueous humour; 2, cornea; 3, lens; 4, canal of Schlemm; 5, ciliary muscle; 6, hyaloid membrane; 7, suspensory ligament of the lens; 8, retina; 9, choroid; 10, sclerotic; 11, optic nerve; 12, optic disc; 13, macula lutea; 14, vitreous humour; 15, canal of Petit; 16, conjunctiva; 17, ciliary process; 18, iris.

3. *The retinal* and innermost coat is composed of nerve elements derived from the spreading out of the optic nerve.

*The refracting media* consist of—

1. The *aqueous humour* a watery fluid which is situated between the cornea in front and the lens behind.

2. *The crystalline lens,* which is situate between the aqueous and

3. *The vitreous humour,* which occupies the posterior part of the eyeball, and is in close contact with the retina.

There are a few details in connection with these coats and media which must be studied. The sclerotic is a very tough layer only slightly elastic, made up for the most part of fibrous tissue. It is thicker around the entrance of the optic nerve and near its junction with the cornea than elsewhere; at these points it is also firmly attached to the underlying structures; in its intermediate portion it is thinner and separated from the choroid by the lymphatic perichoroidal sac.

The optic nerve does not pierce the sclerotic coat exactly at the posterior pole, but rather to the inner side and at a slightly lower level. The optic nerve, as it pierces the sclerotic, breaks up into bundles which pass forwards in small separate compartments. This sieve-like arrangement in the sclerotic is termed the *lamina cribrosa.*

*The cornea* is the direct continuation forwards of the sclerotic; it is not quite circular in shape, as the sclerotic overlaps the cornea above and below more than laterally. In some animals, *e.g.* the dog, this oval shape of the cornea is much more marked. The anterior surface of the cornea is continuous at the corneo-scleral junction with the conjunctiva. The posterior surface is in contact with the aqueous; the substance of the cornea is separated from the aqueous by a layer of epithelium and some elastic fibres comprising the membrane of Decemet.

One other structure must be noted, and that is the canal of Schlemm: it is a lymph channel which runs in a circle at the periphery of the cornea and is indirectly connected with the anterior chamber on the one hand and the ophthalmic veins on the other. The importance of this lymph circulation in certain disorders of the eye will be pointed out later.

*The choroid* coat is essentially a vascular one. It lies between the sclerotic and retina and is firmly adherent to the latter but not to the former. It is composed of two layers; the one next the sclerotic is a meshwork of veins called the vasa vorticosa.

The layer next the retina is made up of fine capillaries, and is called the tunica chorio-capillaris. On the surface of the choroid, next the retina, is a layer of deeply-pigmented cells; strictly speaking, these cells belong to the retina.

The choroid coat, if followed forwards, is found to be continuous with the *ciliary body,* which consists of a series of processes of the ciliary processes, situated around the periphery of the lens; they vary in number between 60 and 80.

The *iris* is also a continuation forwards of the choroid; in its greater part it lies in contact with the anterior surface of the lens and is separated from the cornea by the aqueous humour. At its periphery it is connected with the margin of the cornea by the ligamentum pectinatum iridis. The iris is circular in shape and has a round opening, usually in its centre, called the pupil. Normally, the iris contains a great deal of pigment situated on its posterior surface; the amount varies in different individuals, and it is this variation which gives rise to the difference in the colour of eyes. The anterior surface of the iris has a striated appearance, due to the presence of fine radiating muscular fibres to which the name dilator iridis is given; there is also a circular band of fine muscular fibres arranged around the pupil, which is called the sphincter iridis.

The ciliary muscle lies close to the periphery of the iris and in contact with the sclerotic coat. It forms a circular band of involuntary muscular fibres, the function of which will be described in dealing with the physiology of the eye.

The retina is composed of two portions :—

1. The nervous layer.
2. The pigmentary layer.

It is firmly connected to the choroid coat by the pigment layer, but the inner surface, although moulded on the vitreous, is in no way attached to it. The retina is a transparent membrane firmly fixed at the entrance of the optic nerve; when observed with the ophthalmoscope this area of entrance of the nerve forms a more or less circular, pinkish white disc called the optic disc. A short distance to the outer side of the disc, and in the axis of the globe of the eye, is a small oval spot, lighter in colour than the surrounding retina, called the macula lutea; it has a slightly depressed centre, the fovea centralis.

### The "Qualified Nurse."

### IMPORTANT MEMORIALS TO THE PRESIDENT OF THE LOCAL GOVERNMENT BOARD.

THE following memorial has been presented by the Workhouse Infirmary Nursing Association to Mr. Walter Long, M.P., President of the Local Government Board:—

SIR,—We respectfully beg to lay before you some observations regarding the recommendations made in the Report of the Departmental Committee appointed by you to inquire into the nursing of the sick poor in workhouses.

(Par. 91.) It is proposed to create a new grade of nurse under the title of "qualified nurse." To this recommendation we take objection for the following reasons:—

(Par. 71.) An attempt to train probationers in such workhouses as are defined in the Report as Minor Training Schools—institutions which apparently need not contain more than a maximum of between 60 and 100 sick-beds—cannot fail to be detrimental to the Poor Law Nursing Service, and will inevitably tend to lower the standard of the nursing profession as a whole.

The striking disadvantages of minor training schools would be:—

(*a*) The absence in small workhouses of a sufficiently varied number of cases by which it is possible for a nurse to gain proper experience.

(*b*) The absence of modern treatment and appliances essential to any system of training, and which are usually to be found in small hospitals.

(*c*) The obvious difficulty of establishing in small workhouses the proper tone in regard to patients that ought to be acquired by a nurse at the beginning of her career; and that can only be gained in an institution primarily devoted to the care and cure of the sick.

(Pars. 91, 97, 94 [1]) (*d*) The fact that at the conclusion of one year a probationer holding a certificate from a minor training school would be launched on the nursing world as a "qualified nurse," perfectly free to leave Poor Law work and to take service in connection with small private and district nursing homes, the managers of which may be willing to accept the certificate she holds as qualifying her to undertake, without supervision, responsible nursing duties. By this means a serious danger to the public would be created.

We beg to express our strong opinion that it will be a retrograde step if the Local Government Board consents to sanction the recognition as "qualified nurses" of women who have not received a proper training in the elements of nursing as it is generally understood.

We beg to remain,
Sir,
Yours faithfully,

(*Signed by*)

H.R.H. PRINCESS CHRISTIAN OF SCHLESWIG-HOLSTEIN, Patron of the Workhouse Infirmary Nursing Association.

Miss J. E. Hopper, Matron of Bethnal Green Infirmary.

Miss A. C. Gibson, Matron of Birmingham Infirmary.

Miss Ellen E. Moriarty, Matron of Brentford Union Infirmary.

Miss Louisa Richford, Assistant Matron of Brentford Union Infirmary.

Miss Isabel H. Myles, Matron of Brighton Workhouse Infirmary.

Miss M. Stuart, Lady Superintendent, Brownlow Hill Workhouse Hospital, Liverpool.

Miss Charlotte M. Williams, Superintendent of Nurses, Cardiff Union Hospital.

Miss E. M. Smith, Matron of Central London Sick Asylum, Hendon.

Miss E. C. Barton, Matron of Chelsea Workhouse Infirmary.

Miss M. Rawson, Matron of Chorlton Union Hospital, Manchester.

Miss Elizabeth Stewart, Matron of City of London Infirmary.

Miss M. E. Shipley, Matron of Fulham Infirmary.

Miss H. E. A. Dixon, Matron of Greenwich Union Infirmary.

Miss Louisa Griffiths, Matron of Hackney Union Infirmary.

Miss Katherine Platt, Superintendent of Nursing, Hampstead Workhouse Infirmary.

Miss J. A. Smith, Matron Kingston-on-Thames Infirmary.

Miss Mary Girdlestone, Matron of Crumpsall Workhouse, Infirmary, Manchester.

Miss E. R. Graham, Matron of Mile End Infirmary.

Miss J. E. Styring, Matron of Paddington Infirmary.

Miss S. A. P. Hannaford, Matron of Poplar and Stepney Sick Asylum.

Miss M. M. Hampson, Matron of St. George's Union Infirmary (Fulham).

Miss F. E. Marquard, Matron of St. Giles' Infirmary, Camberwell.

Miss L. Malim, Matron of St. Mary Abbott's (Kensington) Infirmary.

Miss H. E. Little, Matron of St. Mary's (Islington) Infirmary.

Miss Grace Ramsden, Matron of St. Marylebone Infirmary.

Miss A. W. Orchard, Matron of St. Olave's Union Infirmary.

Miss E. J. Moir, Matron of St. Pancras Infirmary.

Miss Annie Sibley, Matron and Superintendent of Nurses, Salford Union Infirmary, Manchester.

Miss Joan Inglis, Matron of Shoreditch Infirmary.

Miss E. Noëmie Armit, Matron of Southwark Union Infirmary, East Dulwich.

Miss Maria Anstey, Matron of Wandsworth and Clapham Union Infirmary.

Miss E. Mowat, Matron of Whitechapel Infirmary.

Miss Helena Gooding, Matron of Woolwich Union Infirmary.

Miss M. E. Jones, Matron of the General Hospital, Birmingham.

Miss C. Elkington, Matron of the Queen's Hospital, Birmingham.

Miss M. Heather-Bigg, Matron of Chelsea Hospital for Women, and Lady Superintendent-elect for Charing Cross Hospital.

Miss Ellen Price, Lady Superintendent, Hospital for Consumption, Brompton.

### THE "QUALIFIED NURSE"—*Continued.*

Miss D. M. Oldham, Matron of Hospital for Epilepsy and Paralysis, Regent's Park.

Miss S. A. Swift, Matron of Guy's Hospital.

Miss Katherine H. Monk, Matron of King's College Hospital.

Miss Annie Barling, Matron, The Infirmary and Children's Hospital, Kidderminster.

Miss Eva C. E. Lückes, Matron of the London Hospital.

Miss A. D. Lucas, late Matron of the London Temperance Hospital.

Miss E. Fisher, Matron of General Infirmary, Leeds.

Miss E. M. Jones, Matron of the Royal Infirmary, Liverpool.

Miss Isabel C. Bennett, Matron of the Metropolitan Hospital.

Miss G. M. Thorold, Lady Superintendent, Middlesex Hospital.

Miss E. C. Vernet, Matron of the National Hospital, Queen Square.

Miss E. Atkins, Matron of the Royal Hospital, Brighton (late Matron of Shoreditch Infirmary).

Miss H. Wedgwood, Matron of the Royal Free Hospital.

Miss Florence Smedley, Matron and Superintendent of Nurses, St. George's Hospital.

Miss E. M. Medill, Matron of St. Mary's Hospital, Paddington.

Miss H. E. G. Hamilton, Matron of St. Thomas' Hospital.

Miss Gertrude Payne, Matron of Hospital for Sick Children, Great Ormond Street.

Miss I. Finch, Matron of University College Hospital.

Miss Katherine Scott, Matron of Sussex County Hospital, Brighton.

Miss Irene Hardy, Matron of the West London Hospital.

Miss M. H. Cave, Matron of the Westminster Hospital.

J. Harley Brooks, M.D., Medical Superintendent of Mile End Infirmary.

F. H. Champneys, M.D., Consulting Physician to the General Lying-in Hospital.

Charles James Cullingworth, M.D.

Florence Nightingale Boyd, M.D.

Arthur E. Chilcott, M.D., Medical Superintendent, St. Pancras Infirmary, Highgate.

W. Pemberton Fooks, M.B. (Cantab.), Medical Superintendent Brentford Union Infirmary, Isleworth.

Timothy Holmes, F.R.C.S., Consulting Surgeon to St. George's Hospital.

F. R. Humphreys, L.R.C.P.

Roland Lee, M.B., B.A., Assistant Medical Officer Cardiff Union Hospital.

John R. Lunn, F.R.C.S.Eng., Medical Superintendent St. Marylebone Infirmary.

Annie McCall, M.D., Director Clapham Maternity.

C. C. McCall, M.B.C.M., late Assistant Medical Officer Cardiff Union Hospital.

Stephen H. Moore, Medical Superintendent Chelsea Infirmary.

A. J. Pepper, M.S.Lond., F.R.C.S., Surgeon to St. Mary's Hospital, Paddington.

E. C. Perry, M.D., Superintendent of Guy's Hospital.

H. Percy Potter, F.R.C.S., Medical Superintendent of Kensington Infirmary.

W. J. Potts, M.D., Medical Superintendent Bethnal Green Infirmary.

M. H. Quarry, M.B., Medical Superintendent Lambeth Infirmary.

Mary Scharlieb, M.D., M.S.Lond.

Thomas D. Savill, M.D.

A. Sheen, M.D., Medical Officer Cardiff Workhouse and Hospital.

Solomon C. Smith, M.D., M.R.C.P.

Guy Neville Stephen, L.R.C.P., D.P.H.Lond.

Maurice Squire, Medical Superintendent Paddington Infirmary.

Dawson Williams, M.D., Editor of the *British Medical Journal.*

Edward T. Wilson, M.B.Oxon., F.R.C.P.Lond. (Cheltenham).

John Williams, M.D.

Miss Agnes M. Alexander, Poor Law Guardian Kensington.

Henry Bonham-Carter, Secretary to the Nightingale Fund.

H. Cosmo O. Bonsor, Treasurer Guy's Hospital.

Edmund Boulnois, M.P.

William Bousfield.

Sir Henry C. Burdett, K.C.B.

Miss S. Cartwright, Secretary to the Registered Nurses' Society.

The Rev. J. Erskine Clarke, President South London District Nursing Association.

Mrs. Bedford Fenwick, President International Council of Nurses; Editor *British Journal of Nurses.*

S. D. Fuller, Poor Law Guardian Paddington.

Mrs. Milner Fothergill.

Miss Griffiths, late Matron of Lambeth Infirmary.

Miss R. P. Fynes-Clinton, Secretary of the Incorporated Midwives' Institute and Trained Nurses' Club.

The Honble. Sydney Holland, Chairman of the London and Poplar Hospitals, Member of Council of Queen Victoria's Jubilee Institute for Nurses, Member of Board of Queen Alexandra's Imperial Military Nursing Service.

The Dowager Lady Jenner.

J. H. Johnstone, M.P., Member of the Association of County Councils.

Mrs. J. H. Johnstone, Superintendent County Nursing Association Sussex.

Miss Kelaart, Poor Law Guardian Bedford Union.

Mrs. Latter, Hon. Sec. Sectional Committee on Nursing of the Midwives' Institute, formerly Matron of Chelsea Infirmary.

Miss Annie Louisa Lee, Hon. Sec. Meath Workhouse Nursing Association.

Miss A. McClure, Poor Law Guardian Rugby Union.

Miss L. Maule, Acting Editor *Nursing Notes.*

Mrs. Malleson.

The Lord Montagu of Beaulieu.

Miss S. Mosley, Poor Law Guardian Hastings Union.

The Rev. Arthur L. B. Peille, President of Queen Victoria's Jubilee Institute for Nurses.

Mrs. Theodore Acland, Hon. Sec. of Queen Victoria's Jubilee Institute for Nurses.

W. G. Rathbone, Hon. Sec. of Queen Victoria's Jubilee Institute for Nurses.

Mrs. Minet, Member of Council of Queen Victoria's Jubilee Institute for Nurses.

Miss Rosalind Paget, Member of Council of Queen Victoria's Jubilee Institute for Nurses. Member of the Midwives' Board.

Miss Pauline W. Peter, General Superintendent of Queen Victoria's Jubilee Institute for Nurses.

Miss Amy Hughes, Superintendent of County Associations Affiliated to Queen Victoria's Jubilee Institute for Nurses.

Mrs. Piggott, Chairman of the Nursing Committee Colonial Nursing Association.

Professor Lane-Poole, M.A., L.T.T.D.

The Rev. C. Darby Reade, M.A., J.P., late Chairman of the Kensington Board of Guardians.

Miss Annie E. Rossiter, Kent Nursing Institute.

Miss E. Vincent, formerly Matron of St. Marylebone Infirmary.

Mr. C. J. Wood, Chairman of Sectional Committee on Nursing of the Incorporated Midwives' Institute.

Miss Margaret Gouldson, Matron Cripples' Home Emscott, Warwick; late Superintendent Nurse Alcester Union Infirmary.

Miss Laura Alloway, Member of the Royal British Nurses' Association.

Miss E. M. Dickson, Member of the Royal British Nurses' Association.

Miss Maud Hollis, Sister Kidderminster Infirmary.

Miss Kate Oxlep, Sister Kidderminster Infirmary.

Miss Edith Scudemore, Sister Kidderminster Infirmary.

Oswald A. Browne, M.D., F.R.C.P.

W. L. Brodie Hall, Poor Law Guardian Eastbourne.

J. F. Akers, Poor Law Guardian Bromley.

W. F. Hicks-Beach.

M. C. L. Campbell, Poor Law Guardian Cheltenham.

A. Cardew, M.R.C.S., Hon. Obstetric Surgeon Cheltenham District Nurses' Association.

H. C. Lawrence, M.D., Hon. Physician Cheltenham District Nurses' Association.

J. F. Crawford, Secretary Victoria Home, Cheltenham District Nurses' Association.

K. M. Bladen, Victoria Home, Cheltenham, Superintendent District Nursing Association.

The Lady Belhaven and Stenton,
Mrs. H. Bonham Carter,
Mrs. S. D. Fuller,
The Hon. Mrs. Hardcastle,
The Viscountess Knutsford,
The Lady Montague of Beaulieu,
Mrs. A. C. Powell,
Miss E. Murray-Smith,
The Lady Wantage,
} Members of the Executive Committee of the Workhouse Infirmary Nursing Association.

The Hon. Mrs. J. G. Talbot, Vice-President of the Workhouse Infirmary Nursing Association, and Chairman of the Executive Committee.

Miss Louisa Twining, Vice-President of the Workhouse Infirmary Nursing Association.

Miss Wilson, Treasurer of the Workhouse Infirmary Nursing Association, and President of the Incorporated Midwives' Institute; Member of the Midwives' Board.

R. V. Gill, Secretary.

6 Adam Street, Adelphi, W.C.
January 17th, 1903.

### SUPPLEMENTARY MEMORIAL.

The Executive Committee of the Workhouse Infirmary Nursing Association have also addressed the following memorial to the President of the Local Government Board:—

SIR,—We, the members of the Executive Committee of the Workhouse Infirmary Nursing Association, beg to lay before you the following objections to:—

(Par. 70) *a.* The proposed reduction in the number of superintendent nurses.

*b.* The recommendations that in workhouse infirmaries containing from 60 to 100 beds the appointment of a superintendent nurse should be optional with Boards of Guardians.

(*a*) If the reduction of superintendent nurses from 227 to 136 is carried out, the standard of nursing organisation of 91 workhouses will probably be in time materially lowered; these workhouses will no longer be subject to the rules of the Nursing Order of 1897 which are now in force, and it is therefore probable that a state of friction will continue. Superintendent nurses will be succeeded by trained nurses, the qualifications (par. 67) of whom are defined, but not their status as regards (1) their responsibilities in relation to the master and the matron, or (2) to the nurses and servants under them, nor even (3) as to the extent of their own duties. Definitions as to these important questions remain as they were laid down in the Order of 1847, at which time trained nurses were practically non-existent. This recommendation appears to have been supported in the Report (3255) by the opinion of one witness only.

(*b*) In relation to pars. 63 (1) and 72 we beg to point out that, considered as a principle, the joint appointment of a workhouse matron as trained nurse or as matron and superintendent nurse may have serious drawbacks, among which we beg to draw attention to the following:—

(Par. 62 [1].) That a matron who acts also as trained nurse may have only "qualified nurses" to carry out her directions, who would themselves be insufficiently trained.

(2) That a matron who also holds office as superintendent nurse could not give the necessary time for training probationers.

(3) That there would be grave danger of conveying infection by a matron whose joint duties would necessarily lead her into all parts of the house, including the sick-wards, and who would frequently be in charge of maternity cases.

We desire further to point out that should a revision of the grant to Guardians under Section 26 (1) of the Local Government Act, 1885, be decided on, it is desirable that a clearer definition should be made of the qualifications of nurses in respect to whose salaries the grant would be paid.

The difficult question of efficient nursing in small workhouses appears to be inadequately dealt with in the recommendations contained in the Report. That the question can be met is shown by reference to the Order of the Irish Local Government Board of July, 1901, which deals with small as well as large workhouses, and which, we understand, is working in a satisfactory manner.

We are, Sir, yours faithfully,

(For the Committee)

MERIEL S. TALBOT, Vice-President, and Chairman of the Executive Committee.
LOUISA TWINING, Vice-President.
T. WILSON, Treasurer.

### A Day in a German Klinik.

BY A CORRESPONDENT.

WHAT mean those far-away sounds? Surely it is not already day, and time to leave one's snug down-covered couch! Blessed reflection. There is yet an hour's grace, the distant sounds merely imply that the goddess of the kitchen has begun her day's work and in an hour will expect both patients and workers to be ready for breakfast. How delightful is that last hour, and with what reluctance does one drag oneself from bed, that nightly refuge of refreshment to the toiler.

### BREAKFAST AND BUSINESS.

Eight o'clock arrives, and down from the upper regions—foreign hospitals have their kitchens at the top of the building—comes the lift laden with breakfasts for nurses and patients. After the pleasure of coffee and sweet rolls follows the business of cleanliness, first of one's patient,

### A DAY IN A GERMAN KLINIK—*Continued.*

then of his room. The rougher part of the work is done by a stalwart German wardmaid. A serious case has the undivided services of a nurse, less serious cases are attended by the house sisters, and only those who have known it can fully describe their loving care and attention.

### The Doctors.

Half-past nine o'clock. Bang goes the great outer door, a sure signal to experienced ears that the first of the five doctors whose patients fill this klinik has arrived. Here he is; a youngish man, brisk, burly, out-spoken, but consumed with interest for his patients; one to be trusted implicitly in spite of his rough exterior. At 11 o'clock a second tempting meal is sent down from above, and at noon all is bustle and preparation for the arrival of the Herr Professor, that genius who, though still young in years, has made for himself a name by his splendid operative skill. From noon till 2 o'clock all are busy, for not only does the professor attend those of his patients who are still lying down, but also any former patients who are well enough to go home and merely need such slight attention as the renewing of bandages, etc. At 2 o'clock appears another type of doctor. Cold, calm, collected, he goes his rounds, disturbing no one, requiring no assistance, reserved and quiet, but with a depth of reassuring strength in his grey eyes that leaves his most nervous patients comforted in spite of their fears and his taciturnity.

### More Meals.

From 2 to 3 busy Sisters flit about with trays of delicious dinners, and at 3 o'clock there is a lull while the workers enjoy their well-earned meal. At 3 arrives yet another physician—a stout, rosy-faced professor, always cheerful, with ever a joke for his patients, who enjoy his visits immensely. Four-thirty brings the rightly-famous German coffee and rolls for the patients, and 7 o'clock their light nutritious supper. The workers have their supper at 8, and after it prepare their patients for the night. By 9.30 a deep peace has fallen upon the klinik, only broken by a softly-treading form or two gliding round in charge of a wakeful or suffering patient.

### Government Control.

Such is my experience of a day in a German private nursing home, or klinik. Perhaps a few particulars as to how the klinik differs from its English equivalent may be of interest. The Weir-Mitchell treatment as practised here rejoices in the appropriate, if uncomplimentary, name of "Mastkur," the verb "mästen" signifying to fatten cattle and pigs for show or sale! It is carried out with true German zeal, and produces often the most marvellous results. In one case which came under my knowledge the patient gained $38\frac{1}{2}$ lbs. in seven weeks! All kliniks, being under Government control, are liable to surprise visits from a medical inspector at any hour of the day. Each one, however small, must have its own operating theatre, for operations are frequently performed in the middle of the night, and each one is equipped with an excellent telephone, attendance on which is no sinecure, as I know from experience. In this particular klinik a concession was made to superstitious patients, and no room was numbered either 5 or 13, both unlucky numbers in Germany. One member of the household must not be forgotten, a handsome black French poodle, "Moor," who gravely made his rounds with his mistress, the Oberin, and cheerfully assumed the rôle of nurse-companion to any patient desirous of his kind services, especially if well enough to include biscuits in his dietary!

### Queen Victoria's Jubilee Institute for Nurses.

Her Majesty Queen Alexandra has been graciously pleased to approve the appointment of the following as "Queen's Nurses," to date January 1st, 1903:—

### ENGLAND.

| Name. | District Training at | Where Serving. |
|---|---|---|
| Susan M. Marsters | Holloway, N. | Hampstead, London. |
| Lizzie Casey | Shoreditch | London. |
| Winifred Farey | Chelsea | " |
| Constance Marian Fuller | Hammersmith | " |
| Jane Edith Highet | Central Home, London | " |
| Edith Lovegrove | Camberwell | " |
| Susie Millsum | Kensington | " |
| Kate Emily Pearson | East London | " |
| Edith Elizabeth Powell | Westminster | " |
| Mary A. Stephens | " | " |
| Jane Yellow | Shoreditch | " |
| Laura Scott | Cardiff | Accrington |
| Kate Helen Steff | Portsmouth | Barnett |
| Janet Steele | Leeds | Bedford (South) |
| Ella Tenniswood | Salford | Birkenhead |
| Amelia Ashford | Birmingham | Birmingham |
| Florence S. Crowther | " | " |
| Adria H. G. Wright | Brighton | Brighton |
| Fanny Evelyn Mellor | Salford | Cheltenham |
| Edith Mabel Gooch | Walworth | Colchester |
| Laura Jackman | Camberwell | Crook |
| Kate Badcock | Shoreditch | Dartmouth |
| Gertrude E. Magson | Chelsea | Darwen |
| Sophie Polsue | Bermondsey | " |
| Elizabeth McClymont | Liverpool | Drighlington |
| Margaret Welch | Gateshead-on-Tyne | Gateshead-on-Tyne |
| Ethel Mary Frith | Cardiff | Gloucester |
| Elizabeth Louise Hill | Bermondsey | " |
| Bessie Emma Hinley | Camberwell | " |
| Eva Bird | Southampton | Handsworth |
| Louise Fawdrey | Shoreditch | Heywood |
| Betsy Flowers Fulcher | Plaistow | Lincs. N. Assn. |
| Kate Helen Huntsman | " | " |
| Kate Turner | " | " |
| Adela Isabella Austin | Liverpool | Liverpool |
| Margaret Agnes Forbes | " | " |
| Susan Stuart Gemmell | " | " |
| Bessie Bridge Henshaw | " | " |
| Katharine Mills | " | " |
| Margaret Roberts | " | " |
| Margaret Jane Thomas | " | " |
| Ellen Harris | Central Home, London | Malvern |
| Mary Jane Baldwin | Manchester | Manchester |
| Ada Kate Howlett | " | " |
| Marie Toomey | " | " |
| Sarah Grayson | Minchinhampton | Minchinhampton |
| Jane McEwen Hutchinson | Salford | Pateley Bridge |
| Jenny Grey | Central Home, London | Penzance |
| Kate Thomas | " | Pontypool |
| Maud M. Macdonald | Portsmouth | Portsmouth |
| Mary Jane White | Hammersmith | " |
| Henrietta R. Whealler | Shoreditch | St. Ives |
| Gwendoline M. E. Miller | Portsmouth | Silvertown, E. |
| Helena Mathieson | Brighton | Southampton |
| Elizabeth J. Clarke | Portsmouth | Sparkhill |
| Annie Heath Brown | Brighton | Stamford |
| Hephzibah Rees | " | Strood |
| Alice F. A. Rowe | Central Home, London | Torquay |
| Lucy Mabel Glass | " | Tottenham |
| Christina N. N. Bell | Dublin | Ulverston |
| Hessie Harrison | Central Home, London | Warrington |
| Frances Jessop | Bermondsey | " |
| Mary Parkin | Stockton-on-Tees | Willington |
| May Willoughby | Liverpool | " |

### WALES.

| Name. | District Training at | Where Serving. |
|---|---|---|
| Phebe Catherine Hughes | Shoreditch | Bala |
| Margaret Griffiths | " | Bangor |
| Elizabeth O. Evans | Cardiff | Llanrwst |
| Mabel Pickthall | Liverpool | Swansea |
| Mary Ellen Jones | Walworth | Towyn |

SCOTLAND.

| Name. | District Training at | Where Serving. |
|---|---|---|
| Matilda Acheson .. .. | Edinburgh .. | Edinburgh |
| Jean Brown .. .. .. | " | " |
| Annie Duncan .. .. .. | " | " |
| Jean Macdonald .. .. | " | " |
| Alice Young .. .. .. | " | " |
| Elsie Pirie .. .. .. | " | Aviemore |
| Isabella Lees .. .. .. | " | Back, Lewes |
| Sara Elizabeth Hayes .. .. | " | Kelty |
| Rebecca Walker .. .. | " | Loch Awe |
| Jean Stewart Hagart .. .. | " | Renton |
| Donatina Macarthur .. .. | " | Sanquhar |
| Margaret Baikie .. .. | " | Thornlie Bank |
| Mary Campbell .. .. .. | " | Tiree |
| Charlotte Morton .. .. | Dundee .. | Dundee |
| Mary Ann Parkinson .. .. | " | " |
| Mary Wall .. .. .. | " | " |

IRELAND.

| Name. | District Training at | Where Serving. |
|---|---|---|
| Mai Carroll .. .. .. | Dublin .. | Dublin |
| Lizzie Daly .. .. .. | Manchester .. | " |
| Constance V. Kirkpatrick .. | Dublin .. | " |
| Violet Laurie .. .. .. | " | " |
| Emily Agatha Martin .. .. | " | " |
| Rosanna Denvir .. .. .. | " | Cushendall |
| Mary Jane Chisholm .. .. | " | Drogheda |
| Elizabeth Ruth Simpson .. | Londonderry | Londonderry |
| Julia O'Callaghan .. .. | Dublin .. | Mount Talbot |
| Mary Agnes Lewis .. .. | " | Newmarket-on-Fergus |
| Lily Storey .. .. .. | " | Newry |

### Everybody's Opinion.

**[Correspondence on all subjects is invited, but we cannot in any way be responsible for the opinions expressed by our correspondents. No communication can be entertained if the name and address of the correspondent are not given as a guarantee of good faith, but not necessarily for publication. All correspondents should write on one side of the paper only.]**

### THE CIVIL HOSPITALS AND THE ARMY NURSING SERVICE RESERVE.

"THE HON. SYDNEY HOLLAND" writes: "Army Sister" asks me whether I would wish to deny to professional nurses the right to volunteer their services for the use of their country, and expect them to be content to be supplied to the War Office? No, I do not wish anything of the sort. Let any nurse volunteer who wishes to do so, but whether she should be allowed to go should depend on her matron's opinion as to her fitness for nursing soldiers, and this I maintain, is the only way to secure good nursing for soldiers. Since my first letter, I have received from the manager of a really good nursing institute an account of the lamentable unfitness of some of the nurses who were taken into the A. N. R. from her staff, which I do not quote, though I have her leave to do so, because these scandals are best forgotten. I repeat that the staffs of the hospitals are the only proper National Reserve, because from the staffs of hospitals alone can you be sure of the best nurses available at the moment, though, as I said before, these could be supplemented by other nurses well known to the matrons of civil hospitals as pre-eminently suitable for this special work. The matrons of the leading hospitals did not disapprove of the formation of this Army Nursing Reserve out of mere cussedness or without very good reasons, and their judgment has been amply justified by results. Exactly what they prophesied has happened. "Reserve Sister" says she will stick to her guns, and will maintain that if the matrons would work for the Reserve by encouraging their best nurses to become members, in a reasonable proportion to the strength of the staff, a very high standard of nurses would be ensured, and the matrons would have practically the selection of the A.N.S.R members. What would be the good of this? Supposing to-morrow the matron of the London Hospital were to go round and select from our nurses, say, 80 of the best, what would be the use? They would be scattered all over the world probably, would be married, have given up nursing, be in private institutions, etc., before war broke out again, and as a "Reserve" would be useless. More than that, being on the Reserve, they would be the first to be called on, and there would be no guarantee from the matron that they had kept up to the standard for which she selected them. "Ah but," I hear the reply, "she need not go to these back numbers, she would have in her hospital a present Reserve." This is exactly the plan I suggest, without doing any hardship to the back numbers. She would when war breaks out ask for volunteers for the War Office, probably every nurse she had would volunteer, and she could select the best fitted for the work. I think it most unlikely that any matrons will change their minds about the futility of the present plan.

"E. J." writes: I think your number for January 10th extremely interesting all round. There is one article in particular I should just like to say a few words about. I am a member of the Army Nursing Service Reserve, having joined at the beginning of the war. When I returned from the "front," four months ago, I applied for the Queen Alexandra's Imperial Military Nursing Service, but was refused on account of my having passed the age-limit—I was 35 years and seven months—and this, although I had been on active service for more than two years. Was that fair? Also, if I were a man, I should be entitled to four bars to my medal; as it is, we poor nursing sisters get neither bar nor clasp, but have to fasten our medal on with a pin. All must allow that our work is quite as hard as most medical officers. I met a civil surgeon on a hospital ship who had never slept off the boat, was making a single voyage, and did not go on shore except for pleasure while the boat was in dock, and yet he would get the South African medal and a bar. There is no doubt, too, as to a certain amount of boycotting of Reserve nurses. Of course it is hard for matrons to lose members of their staff. Is it not true, and increasingly so, in these days, that staff appointments are not kept permanently as they once were? No doubt this is because of the small salaries given, and the narrowness (shall I call it?) of the life. One is surrounded by sickness and suffering, which certainly is very trying to the strongest of us. Who does not remember the terrible tired feeling one gets, and the longing to do nothing but lie down in off-duty hours? This, though the best tonic on occasions, grows into a bad habit. Speaking personally, I know I often found, when I overcame that disinclination and either went for a walk, a steady cycle ride, or a drive on the top of a car, I felt far more refreshed. Nurses should be encouraged to take more open-air exercise or seek recreation and amusement in some way, run a hobby, anything which will provide a contrast and relaxation from their ordinary occupation. At the same time, those who have taken up private work may often wish themselves back to hospital, where one has a chance to keep up to date and be always learning something. But this is a great digression. From my own observation I cannot altogether agree with Mr. Sydney Holland as to the mistake of the Army in forming a Reserve. I have met several of the original members, at the "front," whose work was quite equal to those of us who joined for the war, even those straight from hospital. I enjoyed my time in South Africa immensely, and feel thankful for the experience gained there. Of course we had some hardships, but we should not have known we were on active service if everything had gone smoothly. Taking into account the sudden amount of work thrown upon headquarters, what wonder is it that it was overwhelmed at first? Considering that nearly everybody and everything had to be sent over 5,000 miles of water and then hundreds of miles by rail, in a country where railroads consist of a single line only, I think that things were managed wonderfully well.

### A PLEA FOR THE R.A.M.C.

"X. Y. Z." writes: May I be allowed to say a few words on behalf of that much-abused anomaly Tommy Atkins R.A.M.C.? Starting from the Zulu war to the last Soudan Expedition, all the work in connection with the sick was done by the corps without any civil assistance, and the brunt of the work fell upon Tommy. During these wars and for some time afterwards he was called upon to practically do all the nursing, cleaning, responsibility for equipment, etc., in connection therewith. His duties extended from day to night, and often for protracted periods he had only one night in bed, with what time he could snatch for his meals. All has come the same to him, however, whether cholera dodging in Egypt, enteric fever nursing in all its objectionable phases, contagious fevers of all kinds. Often sick himself, he has stuck to his guns, and for what? Going out as a detail, not like a regiment, "with bands playing, etc.," he comes back a detail, and quietly drops into his place in hospital. Except in individual cases he receives no laudation, no one writes his praises, no society papers extol his virtues; in fact, he is nobody's child. Now as to his position in the new *régime*. He is to divide his work with the female nurse; and I quite agree with your lady correspondents that this appears to be practically impossible. A private's pay on joining the R.A.M.C. is made up as follows:—Regimental pay, 1s. 2d.; corps pay, 4d.; rations, 6d. This gives 14s. a week. The staff nurse receives £30 a year and 15s. a week rations, equalling 27s. a week. Now as one of your correspondents so aptly puts it, "As it is a soldiers' army it should be a soldiers' hospital! Why not then offer the 27s. a week to the male civilian, which would, no doubt, induce the right class to throw in their lot with, and become a Tommy of, the R.A.M.C.?"

### "NURSING BY ORDERLIES."

"WILLIAM GUTHRIDGE, 23 York Place, W.," writes: I should like to thank "A Reserve Sister" for her sensible article on the above subject. "Reserve Sister" has had plenty of experience with male nurses, and what is of more importance, she has the courage to give her opinion about them. To quote her own words: "A good male nurse is not to be surpassed; nor, be it said, is a bad one." One more sentence in her letter is in excellent form, namely, "The employment of male nurses in our Army and Navy is necessary and useful, and could be introduced with the greatest advantage into our civil hospitals." I made a similar suggestion in your issue of April 26th last, and I hope the day will come when male nurses will be trained and at work in all general hospitals. At present the National Hospital, Queen's Square, is the only hospital in this country training male nurses. The training in several well-known asylums is as good and on similar lines. I know nothing about the training of orderlies or what their capabilities are; but judging from what I saw at Bisley Camp in the general hospital there some three years ago I should say there is far too much emphasis laid upon "one, two, three" and "right about turn" for them to make good nurses. The men would be better employed, to their own advantage as well as the patients', if the time were spent in the hospital attending to the patients. The complaints made about the orderlies during the late war were in every way unfair. Numbers of the men must have been pressed into the work practically without any previous training, and then expected to perform wonders. To judge of the male nurses on that basis is absurd. The facts of the case prove the good work done by the orderlies right through the campaign. Take for one instance the epidemic of enteric at Bloemfontein, when 1,000 men were struck down. In such a case the orderlies would be the principal source of help, otherwise one shudders to think of the neglect, as the number of patients and the amount of work to do would be out of all proportion to the number of sisters and nurses on the spot. Dr. Urquhart, of Perth, in his excellent letter, said he only regretted that the male attendants were not given the same opportunities as the female nurses, viz.—to enter the wards of the general hospitals. Your correspondent's report from the meeting of the "German Society of the Red Cross at Elberfeld," mentions "that the subject of the nursing of male patients has of late been thoroughly discussed in all nursing centres. The general opinion among this German assembly was the same as that of most English workers: in hospitals and towns where male help can be obtained the catheter and bathing of patients should not be undertaken by female nurses." All this is as it should be, and tends to show how strong is the feeling on this subject among right-minded persons. Until the committees of our large hospitals decide to train male nurses, the whole thing must remain very much as at present. If male nurses were trained in all general hospitals there would always be a good supply of trained men for unforeseen circumstances, such as the late South African war; the men would volunteer in large numbers on such occasions, and they would go as competent nurses, very different to the present state of things, when they are brought into the military hospitals from the ranks without the slightest knowledge of their work, or how to go about it. Male nurses will be grateful to "Reserve Sister" for her kind appreciation of their work.

### Appointments.

BOROUGH ISOLATION HOSPITAL, LEICESTER.—Miss Irene Webb has been appointed matron. She was trained at the Western Fever Hospital, London, and also at the London Hospital, Whitechapel, E. She was afterwards successively head nurse at the Royal Hospital, Putney; charge nurse at the Western Hospital, Fulham; night superintendent at the Grove Hospital, Lower Tooting, and for nearly three years assistant matron at the Fountain Hospital, Lower Tooting.

BOROUGH SANATORIUM FOR INFECTIOUS DISEASES, SUNDERLAND.—Miss Cecil Bell has been appointed matron. She was trained at Sunderland Infirmary where she has since been night superintendent. She has also been matron at the Knight Memorial Hospital, Blyth.

HARROGATE AND KNARESBOROUGH JOINT ISOLATION HOSPITAL.—Miss L. Morgan has been appointed matron. She was trained at the Royal Infirmary, Newcastle-upon-Tyne, subsequently becoming one of the head nurses. She was afterwards engaged in private work for two years, for more than a year was charge nurse at the Eston Accident Hospital, and for nearly four years matron at the Eston Sanatorium.

KENDAL HOSPITAL.—Miss Elsie Povah has been appointed staff nurse. She was trained at Epsom Infirmary.

KINGSTON UNION INFIRMARY.—Miss C. L. Elliott has been appointed sister to the obstetric wards, and Miss F. A. Selby assistant night sister. Miss Elliott was trained at Birmingham Infirmary, where she was afterwards ward sister for two years. She holds the L.O.S. certificate. Miss Selby was trained at Birmingham Infirmary, and was subsequently ward sister at Bradford Union Hospital. She holds the L.O.S. certificate.

NORTH DEVON INFIRMARY, BARNSTAPLE.—Miss Lillian Fincke has been appointed sister. She was trained at the Royal Albert Hospital, Devonport.

SEAMAN'S HOSPITAL, MADEIRA.—Miss Christine Schliemann has been appointed matron. She was trained at the Royal Hants County Hospital, has since had charge of a home at Ventnor, and also done temporary matron's duty at the Evesham Cottage Hospital. For the last few years she has been attached to the Nurses' Co-operation, 8 New Cavendish Street, and has only recently returned from the Concentration Camps, South Africa.

WHITE OAK SCHOOL, SWANLEY, KENT.—Miss Emeline Lynch has been appointed matron. She was trained at St. Bartholomew's Hospital, Rochester, where she afterwards served as sister for three years. In December, 1896, she was appointed superintendent nurse of the Ophthalmic School, Hanwell, W., and since 1898 has held the post of matron and superintendent of nurses at that institution.

WORKHOUSE INFIRMARY, GREAT GRIMSBY.—Miss Julia Lund has been appointed charge nurse. She was trained at Keighley Union Infirmary, and has since been nurse at the Convalescent Home, Coatham, Redcar.

### "The Hospital" Convalescent Fund.

THE Honorary Secretary acknowledges with thanks the receipt of 2s. 6d. from "Policy 8,337," and of 1s. from Nurse E. C. Rogers.

### Echoes from the Outside World.

### Movements of Royalty.

THE King and Queen returned to London on Tuesday, and, having lunched at Buckingham Palace, proceeded to Windsor, where they will remain for a fortnight. A greeting to the King by Marconi's wireless telegraphy from President Roosevelt on behalf of the American people elicited from his Majesty the following reply by the same means of communication:—"I thank you most sincerely for the kind message which I have just received from you through Signor Marconi's Transatlantic wireless telegraphy. I sincerely reciprocate in the name of the people of the British Empire the cordial greetings and friendly sentiments expressed by you on behalf of the American nation, and I heartily wish you and your country every possible prosperity."

### The South African Settlement.

ALL the other incidents in the visit of Mr. Chamberlain to Johannesburg are of minor interest to the banquet at which he was entertained in the Wanderers' Hall on Saturday evening, and the speech in which he indicated the policy of the British Government as to the future. Having stated that his expectations as to his reception in Johannesburg had been fully realised, and that the interviews he had had with representative Boers had impressed him favourably, the Colonial Secretary, amid prolonged cheering, said that Parliament would be asked to guarantee a loan of £35,000,000 for expenditure in the Transvaal and Orange River Colonies, and that as soon as this has been placed on the market, there would be issued another of £30,000,000 payable in three equal annual instalments. A group of South African financiers had, he informed his audience, undertaken to subscribe the first 10 millions of the second loan, and for the other two they would ask neither commission nor preferential security. The 30 millions would be a war debt. With regard to the labour question, an inter-colonial conference would be held under the auspices of Lord Milner to consider matters of common interest, including native administration and legislation.

### A Remarkable Incident in Morocco.

THE news from Morocco is still grave, and further fighting has been reported between the Sultan's and the Pretender's troops, the former sustaining a reverse. This occasions no surprise to those on the spot, for the command of the expedition is entrusted to various relatives of the Sultan, most of them very incapable. The British Consul, Mr. Macleod, has insisted on the English and American missionaries leaving at once, in view of what might occur later on. A private letter from Fez from some English ladies says: "When we went out on Thursday the people cursed us, saying 'What is this filth here in our street!' On another occasion, when we were distributing medicine, a well-dressed woman and child twice emphatically cursed us, and even a party of mountaineers armed with guns stopped in the middle of the street as we approached, and, cursing us with one voice, passed on. We have been spoken of as 'cursed dogs.'"

### Death of a Great Philanthropist.

MR. QUINTIN HOGG, who died on Saturday morning, in his bathroom from asphyxia, will be mourned by thousands whom he has helped and benefited. He was educated at Eton, where he was well known as an athlete, having been captain of the Old Etonians' Football Club for seven years. As early as 17 he became much impressed with the need of giving a helping hand to the working boys and young clerks of the metropolis, who frequently drifted into evil ways for the want of wholesome recreation and an institution to go to in their spare time where they could both get instruction and amusement. He founded homes for working lads, and also the Youths' Christian Institute in Endell Street, and later on when the Polytechnic, in Regent Street, was closed as a place of amusement, he took it over at a cost of £90,000, and made it the home of his already established institutions. When he started the membership was 2,000, all young men or lads, now there are nearly 18,000 members and students of both sexes.

### A Famous Journalist.

THE fact that the celebrated Paris correspondent of the *Times*, M. de Blowitz, whose death at the age of 78, was announced on Monday, continued at his post until a few weeks before he was attacked by peritonitis, discharging his duties with unimpaired ability and energy long after he was seventy, shows the absurdity of age-limits. M. de Blowitz was a native of Bohemia, and was asked by Laurence Oliphant to temporarily take his place as correspondent of the leading journal before he had even seen a copy of the *Times*. He was well into the fifties when he permanently threw in his lot with journalism, and entered upon the career in which he achieved such marvellous success. His greatest triumph was, perhaps, his disclosure of the terms of the Treaty of Berlin before it was signed. He had interviewed, or been interviewed, by almost every foreign potentate from the Pope to the Sultan, and was personally acquainted with all the distinguished men of the day.

### The Union Jack Club.

DURING the past few weeks there have been several conferences held at the War Office of influential naval and military officials to discuss the advisability of founding a Sailors and Soldiers' Club in London. The outcome of these discussions is that the "Union Jack Club" will be started as soon as possible, at a cost of £50,000, on a site near Waterloo Station. The reason of the selection of this particular locality is that it is computed that no fewer than 100,000 soldiers and sailors arrive at the terminus of the South-Western Railway every year. It is not expected that there will be any necessity to ask Government for a special grant as a large sum of money has been already promised. The institution will contain 400 bedrooms, a restaurant, smoking rooms, billiard rooms, reading room, and other club accommodation, for all of which very moderate sums will be charged. Any member of the Imperial forces will be allowed to make use of the club, and his uniform will be his passport. Those sympathisers who subscribe £100 to the scheme will be entitled to have a brass tablet placed in a room as a permanent memorial of relatives and friends who lost their lives in the war.

### Arts and Crafts Exhibition.

FOR the past year or two nothing like a definite collection of "Arts and Crafts" has been shown in London. This year again, however, the society has an exhibition at the New Gallery, the seventh of its kind. There is some beautiful furniture exhibited by the firm of Morris and Co, of which "A Drawing-room Cabinet of Italian Walnut, inlaid with Holly, Satinwood, and Rosewood" deserves special mention, as also some "Silver-grain Bedroom Furniture, inlaid with Pewter and Blue Wood" exhibited by Messrs. Heal, which has the charm of novelty. The wall paper designed by Mr. Walter Crane, called "Orange Tree," is treated in a highly artistic and decorative manner, and he is apportioned Recess No. 1, in which all the exhibits belong to him. A relief in plaster by Mr. E. M. Pope of a procession of children through a wood, the figures winding in and out of the trees, is very clever; and so is the way in which Mr. Frederick Marriott has treated a panel entitled "St. George and the Dragon." Mrs. Phœbe A. Traquair's four silk-embroidered panels are very fine. Miss Florence Steele has designed a pretty "Silver Yacht Trophy," also a pair of "Silver Buckles" and "A Fish-knife" showing considerable talent.

### For Reading to the Sick.

### THE CONVERSION OF ST. PAUL.

AND as he journeyed . . . suddenly there shined round about him a light from heaven.—*Acts* ix. 3, 4.

What sudden blaze is round him poured,
As though all heaven's refulgent hoard
In one rich glory shone?
One moment—and to earth he falls:
What voice his inmost heart appals?—
Voice heard by him alone.

He hears the meek upbraiding call
As gently on his spirit fall,
As if the Almighty Son
Were prisoner yet in this dark earth,
Nor had proclaimed His royal birth,
Nor His great power begun.

O by those gentle tones and dear,
When Thou hast stayed our wild career,
Thou only hope of souls,
Ne'er let us cast one look behind,
But in the thought of Jesus find
What every thought controls.

As to Thy last Apostle's heart
Thy lightning glance did then impart
Zeal's never-dying fire,
So teach us on Thy shrine to lay
Our hearts, and let them day by day
Intenser blaze and higher.

*Keeble.*

---

Let everyone trace all that is good, all that is blessed, all that is perfect, all that is holy, in every man, to God's sovereign will—to God's eternal love.

This, then, first, is the cause of the steadfastness of the saints; and notice next the means by which God works this end within them. St. Paul tells us most distinctly what the means were in his own case. He says, "When it pleased God . . . to reveal His Son in me." Yes, the sight of that love was the means which wrought in the Apostle's mind the change which grew into steadfastness.

The love which he saw in the face of Christ kindled his love in return. It was a marvel to him that he, the persecutor, the reviler, the blasphemer, could be loved; yet God showed him that he was loved, and the union of these two sights wrought this change within him—he saw himself utterly defiled, and yet he saw himself, though thus defiled, beloved of Christ.

But if you cannot see Christ's love, pray that you may see it. The man who goes on praying will surely have revealed to him some sight of that love; the man who wrestles through the night and says, "I will not let Thee go except Thou bless me;" the man who will pray though he faints, praying against fainting; the man who will say, "Show me Thy love, yea, kindle in me by the sight of Thy love, the love I cannot give Thee;" that man will be steadfast unto the end, and the Lord will not leave him; because in the darkness he confesses his Redeemer, because in the time of drought he puts his trust in Him; that presence which is round about him shall make itself known unto him, and the Lord in his hour of weakness will be his strength and reward.—*Bishop Wilberforce.*

### Notes and Queries.

**The Editor is always willing to answer in this column, without any fee, all reasonable questions, as soon as possible.**

**But the following rules must be carefully observed:—**

1. **Every communication must be accompanied by the name and address of the writer.**
2. **The question must always bear upon nursing, directly or indirectly.**

**If an answer is required by letter a fee of half-a-crown must be enclosed with the note containing the inquiry, and we cannot undertake to forward letters addressed to correspondents making inquiries. It is therefore requested that our readers will not enclose either a stamp or a stamped envelope.**

*Maternity.*

(122) Will you kindly tell me if the training at the East End Mothers' Home is equal to that given at Queen Charlotte's Hospital in preparation for the L. O. S.?—*J. P.*

The training at the East End Mothers' Home is admirable, but of course in a large hospital there is a much greater opportunity for seeing a larger variety of cases.

*Massage.*

(123) I shall be much obliged if you will tell me the fees for learning massage, and also say where I can be taught it.—*Annie H.*

The fees for a course of training vary from £10 10s. to £10. You would find that the course given at the National Hospital for the Paralysed and Epileptic, Queen's Square, Bloomsbury, is excellent.

1. Would it be incorrect for a thoroughly trained masseuse to wear uniform? 2. What is the best way for a masseuse to find employment? 3. Is a really efficient woman likely to succeed? 4. To whom should I apply for a second-hand copy of "Gray's Anatomy?"—*Mayflower.*

1. Uniform is worn by all and sundry, consequently fully-trained nurses wear it only when actually necessary or convenient. 2. By personal recommendations from medical men acquainted with her skill. 3. If she is strong enough to maintain a high standard of efficiency, and clever enough to work on business lines. 4. Most likely you could obtain a second-hand copy of "Gray's Anatomy" from some of the book stalls in the neighbourhood of medical schools.

*South Africa.*

(124) Will you kindly tell me if there are good openings for trained nurses in South Africa—I mean for private work? Would you also say if nursing homes would be likely to do well out there?—*Nurse H.*

There are more than enough nurses in South Africa already, and the cost of the necessities of life is very high.

*Medical Gymnast.*

(125) At the end of my two years' course as medical gymnast I should like to offer my services free at one of the large general hospitals. I would give so many hours daily, at such times as would be most convenient. To whom should I apply—to the medical men or to the matron? Is such an offer likely to be well received?—*J. M.*

To the medical staff. It is quite impossible to say how such an offer would be received.

*Lip-reading.*

(126) *Mrs. G.* will thank the Editor to tell her if he knows of anyone qualified to instruct in "lip-reading." The terms must be moderate.

We cannot recommend individuals, and the terms for this kind of instruction are high. You might obtain particulars from the Training School for Teachers of the Deaf, Castlebar Hill, Ealing, W., as this seems to be the nearest school to you.

*Incurables.*

(127) Will you oblige me by giving the addresses of Homes for Incurables?—*Nurse.*

You will find a full list in "Burdett's Hospitals and Charities."

**Standard Books of Reference.**

**"The Nursing Profession: How and Where to Train." 2s. net; post free 2s. 4d.**
**"Burdett's Official Nursing Directory." 3s. net; post free, 3s. 4d.**
**"Burdett's Hospitals and Charities." 5s.**
**"Hospital Expenditure: The Commissariat." 2s. 6d.**
**"The Nurses' Dictionary of Medical Terms." Cloth, 2s.; leather, 2s. 6d. net.; post free, 2s. 8d.**
**"Burdett's Series of Nursing Text-Books." 1s. each.**
**"A Handbook for Nurses." (Illustrated). 5s.**
**"The Physiological Feeding of Infants." 1s.**
**"The Physiological Nursery Chart." 1s.; post free, 1s. 3d.**
**All these are published by the SCIENTIFIC PRESS, Ltd., and may be obtained through any bookseller or direct from the publishers, 28 and 29 Southampton Street, London, W.C.**

## Figures and Tables

**Fig. 1. f1:**